# Genome sequencing reveals high diagnostic yield in children with severe sporadic developmental language disorder

**DOI:** 10.1038/s41431-026-02214-7

**Published:** 2026-08-17

**Authors:** Milou G. P. Kennis, Leenke van Haaften, Karen van Hulst, Rolph Pfundt, Nicole de Leeuw, Jeroen van Reeuwijk, Robin Wijngaard, Lisenka E.L.M. Vissers, Han G. Brunner, Simon E. Fisher, Tjitske Kleefstra, Lot Snijders Blok

**Affiliations:** 1 Department of Human Genetics, Radboud University Medical Center, Nijmegen, The Netherlands; 2 Research Institute for Medical Innovation, Radboud University Medical Center, Nijmegen, The Netherlands; 3 Department of Rehabilitation, Amalia Children’s Hospital, Donders Institute for Brain, Cognition and Behaviour, Radboud University Medical Center, Nijmegen, The Netherlands; 4 Language and Genetics Department, Max Planck Institute for Psycholinguistics, Nijmegen, The Netherlands; 5 Donders Centre for Neuroscience, Donders Institute for Brain, Cognition and Behaviour, Radboud University, Nijmegen, The Netherlands; 6 Department of Clinical Genetics, Erasmus MC University Medical Center, Rotterdam, The Netherlands

**Keywords:** Genetics research, Clinical genetics, Neurodevelopmental disorders, Genetic testing, Rare variants

## Abstract

A developmental language disorder (DLD) is a neurobiological condition characterized by impaired language development despite adequate linguistic input and normal intelligence. The role of monogenic causes in the pathogenesis of DLD is largely unknown and diagnostic genetic testing is rarely offered. This study assessed the diagnostic yield of genome sequencing in severe DLD and investigated the underlying genetic foundations. We designed a prospective cohort study involving trio-based genome sequencing in 25 individuals (aged 3–25 years) with severe DLD, no intellectual disability (non-verbal IQ ≥ 70), no autism spectrum disorder diagnosis and no first-degree family history of DLD or neurodevelopmental disorders. (Likely) pathogenic variants were found in nine of 25 individuals, reflecting a molecular diagnostic yield of 36%. The genetic causes were diverse and involved seven autosomal dominant disorders, one autosomal recessive disorder, and one sex chromosome aneuploidy. In seven individuals, the disease-causing variant occurred de novo. All identified (likely) pathogenic variants were located in genes or loci that have been previously implicated in neurodevelopmental disorders with variable clinical presentations. Importantly, the genetic diagnosis provided actionable insights for counselling and clinical follow-up for all nine individuals. This study reveals a high prevalence of rare (mainly de novo) genetic variants in individuals with severe and sporadic DLD, and demonstrates extensive molecular overlap with other neurodevelopmental disorders. Our results support the implementation of routine trio-based genetic testing in individuals with severe and sporadic DLD.

## Introduction

A developmental language disorder (DLD) is a neurodevelopmental disorder (NDD) that significantly affects a child’s ability to understand and use language, with long-term consequences for academic achievement, social development and overall well-being [1, 2]. With an estimated prevalence of ~6-7% in children, DLD imposes a large societal burden [3, 4]. Despite its high prevalence and impact, the genetic bases of DLD remain poorly understood. In this study, we aim to investigate the role of monogenic (and chromosomal) causes in children with severe and sporadic DLD, in order to better understand its molecular background and to determine the value of genetic testing in this context.

DLD comprises a heterogeneous spectrum of persistent receptive and/or expressive language difficulties across various language domains (e.g. semantics, syntax, and pragmatics), in varying degrees of severity. Terminology for language disorders has changed over time; the term *developmental language* *disorder* (DLD) has replaced previously used terms such as *specific language impairment* and *developmental dysphasia* [5]. Importantly, DLD is distinct from disorders that solely affect speech (e.g. *speech sound disorder* or *childhood apraxia of speech (CAS)*), although these conditions can co-occur. Although no internationally standardized diagnostic criteria for DLD currently exist, there is broad consensus that the term should be applied to children with persistent language difficulties (often lasting into middle childhood and beyond) that have a significant impact on social interaction and educational progress [5]. In the Netherlands, a DLD diagnosis is based on the result of standardized language assessments and can be made from the age of three by a speech and language therapist (SLT) [6, 7]. Crucially, DLD is not diagnosed when the language delays are judged to be a secondary effect of broader NDDs, such as autism spectrum disorders (ASD) or intellectual disability (ID). Likewise, a primary cause like hearing loss or acquired brain injury rules out a DLD diagnosis.

It is well established that genetic factors play a significant role in the development of DLD. Twin studies have reported high heritability estimates (at least 0.5) [8–10], and linkage and genome-wide association studies (GWAS) have identified loci associated with atypical language development (as reviewed by Mountford et al. [11]). Traditionally, most studies on the genetics of DLD have focused on multifactorial or polygenic inheritance models, in which the pathogenesis involves the combined effects of multiple common polymorphisms, each with a small individual effect and distributed across the genome [12]. Moreover, GWAS studies are usually applied to large, clinically heterogeneous cohorts, hampering the identification of rare causes for specific phenotypes. Over time, advances in molecular genetic technologies have provided growing evidence for rare genetic causes in a subset of individuals with DLD [13, 14]. This is demonstrated by cohort studies showing an enrichment of sex chromosome aneuploidies and copy number variants (CNVs) in language disorders [15–17]. These cohort studies thus mainly focused on chromosomal abnormalities, consequently missing single nucleotide variants (SNVs) and indels [15–18], or lacked trio-based designs needed to systematically identify de novo variants [19, 20]. Monogenic causes in DLD remain understudied compared to other developmental disorders such as ID, ASD, and severe speech disorders, in which rare de novo variants are frequently identified [21–24]. This underscores the need for prospective studies involving trio-based exome or genome sequencing in DLD.

Investigating rare monogenic and chromosomal causes of DLD may provide valuable insights into the biological mechanisms underlying atypical language development. Moreover, knowledge on the diagnostic yield of genetic testing in a DLD cohort is crucial to enable evidence-based decisions regarding its implementation in a diagnostic setting. We hypothesize that a substantial proportion of severe and sporadic DLD is attributable to rare (de novo) variants. To test this hypothesis, we conducted a prospective cohort study using trio-based genome sequencing in children and adolescents with severe and sporadic DLD.

## Materials and methods

### Study cohort

This prospective single center cohort study was conducted at the Radboud University Medical Center in the Netherlands between January 2020 and August 2024. The study protocol was approved by the Medical Ethics Review Committee (METC) Oost-Nederland (number 2018-4783). Participant recruitment was enabled by the distribution of a brief study description through Dutch DLD patient organizations, SLTs and clinical geneticists. Interested families could register via a dedicated study website.

Participants had to meet the following inclusion criteria: a diagnosis of DLD established in the Netherlands; age 3–25 years at inclusion; non-verbal IQ ≥ 70; an indication for special education or ambulatory support in the ‘Cluster-2’ setting (support services for children with severe communication impairments in the Netherlands); and a negative first-degree family history for DLD and global developmental delays. The following exclusion criteria applied: a diagnosis of ASD; previous exome or genome sequencing for DLD or developmental disorders; and the unavailability of biological parent(s) for genetic testing. In accordance with the definition of DLD, participants could not have hearing loss that could explain their language impairment.

Clinical data were collected during a study-specific intake visit at the outpatient clinic of the Amalia Children’s Hospital in the Radboud University Medical Center. Clinical information on development (including cognitive, speech and language, and motor development), behavioral difficulties, and comorbidities was obtained from specialized multidisciplinary assessment reports from expert ‘Cluster 2’ centers. Additional information, including a detailed medical history and family history, was derived from parental reports and existing clinical records. During the intake visit, a physical examination was performed by (or under direct supervision of) a clinical geneticist, prospectively assessing growth parameters and facial dysmorphology. All clinical data were stored in a Castor EDC research database.

### Speech and language data

For all participants, reports on prior speech and language assessments and therapy were reviewed by two academic SLTs. Speech and language data were extracted from the first SLT report confirming the diagnosis of DLD. Data from various speech and language assessments were collected, including Reynell Test for Language Comprehension, Schlichting Test for Language Comprehension, Peabody Picture Vocabulary Test (Dutch Version), Schlichting Test for Language Production, Clinical Evaluation of Language Fundamentals 4-Dutch Version (CELF-4-NL), Language Tests for Children, Language proficiency test for all children, and Test instrument developmental language disorders for children aged 4 to 10 (T-TOS). All language tools described assess comparable domains, and raw scores can be converted to standard deviation scores. Language impairment severity was categorized as follows: mild (1-1.5 SD below the mean), moderate (1.5-2 SD below the mean), and severe ( ≥ 2 SD below the mean). Severity scores of both receptive and expressive language impairment were provided, focusing on the following domains: semantics, morpho-syntax, and pragmatics. In addition, severity scores were assigned for both speech perception and speech production disorders. When reported in the SLT file, a speech production diagnosis was documented (i.e. phonological disorder, CAS, dysarthria, phonetic disorder, stuttering, cluttering).

### Genome sequencing and data analysis

Genome sequencing was performed as previously described [25]. In brief, DNA was extracted from blood and the Illumina DNA PCR-free protocol (Illumina, San Diego, CA, USA) was used for library preparation. DNA was tagmented to an average insert size of 450 bp. Genome sequencing was performed on an Illumina NovaSeq 6000 instrument to an anticipated minimal 30-fold coverage, using 150 bp paired-end short-reads.

Read alignment (to GRCh38) and (de novo) variant calling were performed using in-house pipelines as described previously [26]. In short, reads were aligned with the human reference genome (GRCh38) using BWA (version 2.2.1) and the quality of the resulting BAM file was assessed using Qualimap (version 2.2.1). De novo variants were called as described previously [27] and using DenovoCNN. SNVs were called using GATK (version 3.8) and annotated, including Variant Effect Predictor annotations, Gencode basic (Human release 34) gene annotations, frequency information from gnomAD (v3.1.2) and an in-house database, in-house gene panel information, CADD (v1.6), spliceAI and OMIM annotations. Structural variants (SVs) were called using Manta, and CNVs using Canvas, and both were annotated using frequency information from X1000G, Decipher (version GRCh38, https://www.deciphergenomics.org/about/downloads/data), DGV (version 2020-02-25) and an in-house pipeline.

### Variant filtering and interpretation

Variants were filtered and prioritized based on variant quality, variant type, mode of inheritance, allele frequency, conservation, and predicted consequences. To reduce the risk of incidental findings, single-gene variants affecting 59 medically actionable genes (ACMG SF v.2.0 guidelines) were removed before reviewing the data [28]. The initial data analysis was limited to a subset of 1649 genes involved in NDDs (in-house gene panel ‘intellectual disability/developmental disorders’, DG-3.5.0) [29], and a genome-wide CNV analysis. Interpretation of SNVs/indels was initially focused on exonic variants and variants within 8 base pairs of exon-intron junctions. If no (likely) pathogenic variants were identified, an analysis of exonic variants outside the gene panel was performed. In addition, a screening was performed for de novo non-coding variants (SVs and SNVs). In this context, all de novo SVs were assessed. For de novo SNVs, screening was limited to variants that were linked to genes within the gene panel, absent from gnomAD, and likely disrupted splicing (using spliceAI scores) or known regulatory elements (e.g. using ReMM scores).

### Test results and outcome assessment

Individual sequencing results were classified following the ACMG guidelines [30] and reported according to the HGVS and ISCN 2024 nomenclature. The variants were discussed in a multidisciplinary team to evaluate their clinical relevance. Participants with potentially clinically relevant genetic findings were re-contacted to discuss the results and to undergo confirmatory testing at our in-house accredited genome diagnostic laboratory. Clinical follow-up was initiated where needed.

The overall diagnostic yield was calculated by dividing the number of individuals with a (likely) causative genetic finding by to the total cohort size. Additionally, association analyses were performed to explore relationships between specific clinical characteristics and genetic testing outcome. Given the limited sample size, multivariable regression analyses were not feasible. Consequently, individual variables were compared between individuals with and without a genetic diagnosis. Fisher’s exact tests were used to compare nominal values, while mean total IQ scores were compared using an independent samples t-test. *P*-values were adjusted for multiple testing using the Bonferroni correction.

## Results

### Clinical characteristics

A total of 25 individuals, aged 4-24 years, were included in our study (Table 1). Details on the inclusion process and clinical characteristics of the study cohort are provided in Fig. S1 and Table S1, respectively. All participants were diagnosed with a severe form of DLD, as demonstrated by the indication for special education or ambulatory support in the Dutch ‘Cluster-2’ setting. Most individuals (17/25) had a mixed receptive-expressive language disorder (i.e., both language production and comprehension were impaired), while a smaller group (8/25) only had expressive language difficulties. This is consistent with findings from other studies, which report that most children with a language disorder present with a mixed language profile [31, 32], A subset of the cohort also showed speech production disorders (17/25) and/or speech perception disorders (10/25), consistent with previous studies reporting speech problems in children with DLD [33, 34]. Nine individuals were diagnosed with a specified speech disorder, such as phonological disorder (*n* = 9), stuttering (*n* = 2) and/or CAS (*n* = 2). The number of formal speech disorder diagnoses in our cohort may represent an underestimation, because of assessment bias related to the retrospective extraction of clinical data and/or limited diagnostic differentiation in the original clinical reports. The mean non-verbal IQ was 96 (range: 76-128), and six individuals had a delay in acquiring gross motor skills. According to parental reports, 12/25 individuals had behavioral difficulties, including ADHD or attention problems, sensitivity to stimuli, and challenges with emotion regulation. A subgroup of the cohort had medical comorbidities, including congenital abnormalities (3/25), vision problems (refractive errors) (3/25), and abnormal growth parameters (9/25). Recurrent ear infections were common (11/25).

**Table 1:** Cohort characteristics (*n* = 25).

Baseline characteristics	
Age at inclusion, *median (range)*	10.3 yrs (4.2–24.3 yrs)
Age at DLD diagnosis, *median (range)*	3.8 yrs (2.1–11.4 yrs)
Male / Female, *n (%)*	14 (56%) / 11 (44%)
Development	
Total non-verbal IQ, *mean (range)*	96 (76–128)
Gross motor delay, *n (%)*	6 (24%)
Behavioral difficulties, *n (%)*	12 (48%)
AD(H)D, *n (%)*	2 (8%)
Speech/language phenotype	
Expressive language disorder, *n (%)*	25 (100%)
Receptive language disorder, *n (%)*	17 (68%)
Speech production disorder^a^, *n (%)*	17 (68%)
Speech perception disorder, *n (%)*	10 (40%)
Medical comorbidities	
Congenital abnormalities, *n (%)*	3 (12%)
Abnormal growth parameters^b^, *n (%)*	9 (36%)
Skeletal problems, *n (%)*	3 (12%)
Vision problems, *n (%)*	3 (12%)

^a^Nine individuals had a specified speech disorder diagnosis: phonological disorder (*n* = 9), stuttering (*n* = 2), CAS (*n* = 2), cluttering (*n* = 1), phonetic disorder (*n* = 1). ^b^Abnormal growth parameters (≥+2 SD or ≤-2 SD): tall stature (*n* = 1), short stature (*n* = 2), overweight (*n* = 5), underweight (*n* = 1), macrocephaly (*n* = 1), microcephaly (*n* = 2).

After applying strict inclusion criteria, this cohort included both individuals with relatively isolated DLD and individuals with additional congenital abnormalities, abnormal growth parameters and/or dysmorphic features, reflecting the phenotypic heterogeneity of the DLD population.

### Diagnostic yield

In nine of the 25 individuals, genome sequencing revealed pathogenic or likely pathogenic variants, resulting in an overall diagnostic yield of 36%. These variants involved SNVs/indels (*n* = 4 individuals), CNVs (*n* = 4 individuals), and a chromosomal aneuploidy (*n* = 1 individual). In seven individuals, the variant occurred de novo. Additionally, a variant of uncertain significance (VUS) was identified in four individuals, three of whom also had a (likely) pathogenic variant. We found no relevant variants in non-coding regions that could be classified as likely disease-causing or as high confidence candidates. Clinical interpretation of the variants is described subsequently; detailed variant information is provided in Tables 2 and S2, visualization of CNVs/SVs in Fig. S2, and individual case reports in the Supplemental note.

**Table 2:** Genetic variants identified in the DLD cohort.

Ind.	Class	Type	Gene/ Locus	Variant	Associated disorder in the individual
**1**	P	SNV	NFIX	NM_001365902.3:c.143 T > A p.(Met48Lys), dn	Malan syndrome (AD) [OMIM #614753]
**2**	P P	SNV SNV	PAH PAH	NM_000277.3:c.898 G > T p.(Ala300Ser), pat NM_000277.3:c.1042 C > G p.(Leu348Val), mat	Phenylketonuria / Hyperphenylalaninemia (AR) [OMIM #261600]
	VUS	Dup	6q14.3	Seq[GRCh38] 6q14.3(85542954_86446473)x3, dn	N/A
**3**	P	Del	16p11.2	Seq[GRCh38] 16p11.2(29639269_30188288)x1, dn	16p11.2 microdeletion syndrome (AD) [OMIM #611913]
**4**	P	Del	2p16.3	Seq[GRCh38] 2p16.3(47670952_47828053)x1, dn	Intellectual developmental disorder with dysmorphic facies and behavioral abnormalities (AD) [OMIM #618089] + Lynch syndrome (AD) [OMIM #614350]
**5**	P	SV	ChrX	Seq (X)×3	Triple X syndrome [43]
**6**	LP	SNV	TRRAP	NM_001375524.1:c.9479 A > G p.(Glu3160Gly), dn	Developmental delay with or without dysmorphic facies and autism (AD) [OMIM #618454]
**7**	LP	Indel	TAF4	NM_003185.4:c.510dup p.(Gly171fs), dn	Intellectual developmental disorder, AD 73 (AD) [OMIM #620450]
	VUS	Indel	FBRSL1	NM_001367871.1:c.618_619del p.(Cys206*), dn	N/A
**8**	LP	Dup	14q11.2	Seq[GRCh38] 14q11.2(20978759_21508137)x3, dn	14q11.2 microduplication syndrome (AD) [38]
**9**	LP	Dup	2q13q14.1	Seq[GRCh38] 2q13q14.1(110635098_112351943)x3, mat	2q13 duplication syndrome (AD) [40]
	VUS	Dup	10p15.3	Seq[GRCh38] 10p15.3(861923_1384877)x3, dn	N/A
**10**	VUS	SNV	CHD9	NM_001308319.2:c.2242 C > T p.(Arg748*), dn	N/A

*del* deletion, *dup* duplication, *dn* de novo, *indel* insertion/deletion, *LP* likely pathogenic, *mat* maternally inherited, *N/A* not applicable, *pat* paternally inherited, *P* pathogenic, *SNV* single nucleotide variant, *SV* structural variant, *VUS* variant of uncertain significance.

When examining specific clinical characteristics in relation to genetic testing outcomes, we observed that individuals with a genetic diagnosis had significantly lower total IQ scores than those without a genetic diagnosis (mean IQ 84 vs. 102 respectively; *p* = 0.036, independent samples t-test) (Table S3). No statistically significant differences were observed for the other variables, including medical comorbidities, facial dysmorphisms, and specified speech disorders. However, a robust evaluation of these associations would require regression-based analyses on a larger sample size.

### Genetic results

Seven individuals were diagnosed with an autosomal dominant disorder resulting from a (likely) pathogenic variant. Individual 1 had a pathogenic de novo missense variant (p.(Met48Lys)) in *NFIX*, previously reported as causative for Malan syndrome (OMIM #614753), a developmental disorder associated with postnatal overgrowth. This diagnosis is consistent with the individual’s clinical features, including a head circumference of +3 SD. Individual 3 had a pathogenic de novo 16p11.2 deletion ( ~ 550 kb, BP4-BP5), corresponding to the recurrent 16p11.2 microdeletion syndrome (OMIM #611913), a disorder commonly associated with language problems [35]. Individual 4 carried a pathogenic de novo 2p16.3 deletion ( ~ 150 kb) encompassing *FBXO11* and *MSH6*. Haploinsufficiency of *FBXO11* causes a developmental disorder (OMIM #618089), explaining her DLD phenotype. Since the deletion also includes *MSH6*, she was additionally diagnosed with Lynch syndrome (OMIM #614350), a hereditary cancer predisposition syndrome. In individual 6, we found a likely pathogenic de novo missense variant (p.(Glu3160Gly)) in *TRRAP*. Variants in this gene have been reported to cause developmental disorders with variable severity (OMIM #618454). The variant is located within an important protein domain, affects a highly-conserved amino acid, is absent from control databases, and is predicted to be pathogenic by multiple in-silico prediction tools. We consider this a plausible explanation for the individual’s DLD and hypotonia. Individual 7 had a likely pathogenic de novo frameshift variant (p.(Gly171fs)) in *TAF4*. Haploinsufficiency of *TAF4* is associated with a developmental disorder (OMIM #620450), characterized by intellectual disability, abnormal behavior and facial dysmorphisms. The individual’s features, including DLD, anxiety, borderline-low IQ (79) and mild facial dysmorphisms, fit within the phenotype of *TAF4*-related disorders. This individual also carried a de novo truncating variant (p.(Cys206*)) in *FBRSL1*, a gene recently implicated in a syndromic malformation and intellectual disability syndrome [36, 37]. This variant was classified as a VUS, because it does not affect the two N-terminal isoforms implicated in the disorder. In individual 8, we identified a likely pathogenic de novo 14q11.2 duplication ( ~ 530 kb), encompassing 15 protein-coding genes. Overlapping duplications in this region (with varying breakpoints) have been reported before as causative for developmental disorders [38, 39]. Individual 9 carried a likely pathogenic maternally inherited 2q13q14.1 duplication ( ~ 1.7 Mb), a locus that has been curated by the Clinical Genome Resource as ‘emerging evidence for triplosensitivity’ with a broad spectrum of developmental disorders as the associated phenotype [40]. The same individual also had a de novo duplication at 10p15.3 ( ~ 520 kb), which has not been reported before and was classified as a VUS.

One person in our cohort (individual 2) was diagnosed with an autosomal recessive disorder. This individual carried two pathogenic compound-heterozygous missense variants (p.(Ala300Ser) and p.(Leu348Val)) in *PAH*, a gene associated with phenylketonuria (PKU) and hyperphenylalaninemia (HPA) (OMIM #261600). Despite normal newborn screening results, current metabolic testing revealed elevated phenylalanine levels consistent with mild PKU or HPA. Although a causal link to DLD cannot be determined with full certainty, developmental and language problems can result from PKU or HPA [41]. The individual also carried a de novo 6q14.3 microduplication ( ~ 900 kb), classified as a VUS. The duplicated region includes *SYNCRIP*, a gene recently implied in NDDs [42], but evidence for triplosensitivity is currently lacking.

In one participant (individual 5), a sex chromosome aneuploidy (47,XXX) was identified, resulting in a diagnosis of triple X syndrome. This neurodevelopmental disorder, with variable expression, has previously been associated with specific language impairment and is likely to have contributed to the clinical features in this individual, including DLD and social difficulties [15, 43].

Lastly, in one individual (individual 10), we identified a VUS in a gene not yet associated with any specific disorder. This involved a de novo nonsense variant (p.(Arg748*)) in *CHD9*. Although *CHD9* is not yet formally linked to a phenotype, emerging evidence from previous cohort studies [44–47] and correspondence with GeneMatcher collaborators suggests a role for (de novo) *CHD9* variants in developmental disorders.

Importantly, in all nine individuals with a (likely) pathogenic finding, the genetic diagnosis had relevant clinical implications (Supplemental note). For the seven individuals with an autosomal dominant disorder, there is a recurrence risk of 50% for possible future offspring. Furthermore, four individuals were referred to a specialized pediatrician for medical care and follow-up. In five cases, diagnosis-specific tests were recommended, including cardiac ultrasound (*n* = 3), ophthalmological examination (*n* = 1), metabolic blood tests (*n* = 1), and cancer screening (*n* = 1). Also, two families were informed about national expertise centers for their child’s disorder, offering specialized guidance and contact with other affected families.

## Discussion

In this study, we report a high prevalence of rare genetic variants among individuals with severe and sporadic DLD in the absence of intellectual disability. The study is marked by a prospective design and a carefully selected cohort with deep phenotypic characterization. We found an overall diagnostic yield of 36% by identifying a (likely) genetic diagnosis in nine out of 25 individuals. A lower total IQ score was associated with a higher likelihood of a genetic diagnosis. The diagnoses involved seven autosomal dominant disorders, one autosomal recessive disorder, and one sex chromosome aneuploidy, all of which have previously been associated with broader NDD phenotypes. The disease-causing variant occurred de novo in seven individuals, underscoring the importance of rare de novo variants in severe and sporadic DLD. This study shows that severe and sporadic DLD may be the first presenting clinical feature of a broader genetic neurodevelopmental syndrome. We recommend the implementation of trio-based exome or genome sequencing in severe and sporadic DLD in clinical practice, using a broad NDD gene panel (or HPO-based strategy) and including a genome-wide CNV analysis.

In addition to our study findings, one individual (ind. 11) received a genetic diagnosis outside of the study analysis. Trio-based exome sequencing, performed simultaneously with our study in a clinical setting, revealed two pathogenic compound-heterozygous variants in *BRCA2* (Table S2), establishing a diagnosis of Fanconi anemia type D1 (OMIM #605724). The diagnosis was compliant with the individual’s clinical features, including microcephaly, short stature and skin pigmentation anomalies. The variants were not identified within our study since SNVs in genes from the ACMG SF v2.0 list were removed prior to analysis as part of our study protocol (see Materials and Methods). Although this diagnosis is not included in our study results and diagnostic yield, it warrants mention as it likely accounts for the individual’s DLD.

Interestingly, while this study focused on a specific DLD cohort, all identified pathogenic and likely pathogenic molecular causes corresponded to known genetic syndromes affecting multiple developmental domains (e.g., motor skills, speech and language, cognition, and social-emotional development). The genetic diagnoses were diverse, ranging from recurrent and relatively nonspecific disorders (e.g., triple X syndrome) to rare and more syndromic conditions (e.g., Malan syndrome). The identified causes did not appear to share a common biological pathway. Notably, none of the genetic diagnoses were exclusively associated with language impairments, although speech and language difficulties are prominent features of some conditions, such as the 16p11.2 deletion syndrome; [48–50] and sex chromosome aneuploidies [15, 16]. The diagnostic yield of 36% observed in this DLD cohort is comparable to that reported in other developmental disorders [51] and severe speech disorders like CAS [23, 52, 53]. The spectrum of molecular diagnoses is also comparable to that found in other developmental disorders, such as ID or global developmental delays. These findings suggest that (at least a subgroup of) DLD may not reflect a distinct etiological entity, but is rather part of the broader spectrum of NDDs.

Some genetic conditions identified in our cohort may present with non-specific features or reduced penetrance (e.g. triple X syndrome), which could raise questions about causality. However, we consider these findings to reflect the molecular landscape underlying DLD, characterized by genetic heterogeneity and variable phenotypic expressivity. In the nine individuals with a molecular diagnosis, the reported variants were extremely rare, classified as (likely) pathogenic based on variant characteristics, and affected genes or loci previously implied in developmental disorder phenotypes. It is therefore highly plausible that these variants are causal for, or substantially contributory to, the development of DLD in these individuals.

Notably, for all individuals in our cohort with a genetic diagnosis, the findings provided valuable insights for genetic counselling, clinical care and follow-up. Families appreciated the clarity the diagnosis offered, as it gave an explanation for the severe language disorder in their child. Additionally, for the seven individuals diagnosed with an autosomal dominant condition, this implied a significant recurrence risk of 50%. In five individuals, the genetic results had direct clinical implications, including referral to a specialized pediatrician or diagnosis-specific screening recommendations (e.g., cardiac ultrasound). While molecular diagnoses are crucial for these reasons, clinical diagnoses like DLD also remain essential in guiding care. We argue that a genetic diagnosis should not exclude a clinical diagnosis of DLD, but that both should rather coexist.

While our study offers valuable insights into the genetic underpinnings of DLD, we acknowledge the small cohort size (*n* = 25). The limited cohort size primarily reflects the rarity of eligible individuals meeting the inclusion criteria for this selected study population (Fig. S1). Future studies with larger DLD cohorts are needed to validate our findings, define the full molecular landscape, and assess the generalizability of our results to the broader DLD population, including milder and familial forms. Our observation that lower total IQ scores were associated with a higher likelihood of a genetic diagnosis should be replicated in larger cohorts, alongside further investigation of additional predictive variables, as have been studied in detail for CAS [52]. Further research is also required to determine the role of the variants of uncertain significance identified in this study, including the variant in *CHD9*. Finally, it would be valuable to further compare language profiles across different genetic syndromes [54]. Such research could provide important insights into the biological processes and pathways underlying atypical language development, while also helping to inform and refine future speech and language therapy interventions.

In conclusion, this study demonstrates a high prevalence of rare, mainly de novo genetic variants in individuals with severe and sporadic DLD, with direct implications for clinical care and follow-up. The molecular background in our cohort strongly overlaps with that of other neurodevelopmental conditions, suggesting that in these cases, DLD can be regarded as a clinical phenotype within the broader spectrum of NDDs. Overall, our findings underscore the importance of implementing genetic testing into the routine diagnostic evaluation of individuals with severe and sporadic DLD.

## Supplementary information

Supplemental material

Table S1: Clinical characteristics

Table S2: Variant characteristics

Table S3: Associations between clinical characteristics and genetic outcome

## Data Availability

There are restrictions to the availability of raw individual data due to privacy concerns. Pseudonymized individual data generated in this study are available from the corresponding author upon reasonable request.

## Web resources

ClinVar, https://www.ncbi.nlm.nih.gov/clinvar/. GeneReviews, https://www.ncbi.nlm.nih.gov/books/NBK1116/. GnomAD, https://gnomad.broadinstitute.org/. OMIM, https://www.omim.org. UCSC Genome Browser, https://genome.ucsc.edu/.
