# Supplementary material for: Genome sequencing reveals high diagnostic yield in children with severe sporadic developmental language disorder
Source: Eur J Hum Genet. 2026 Aug 17;34(11):1739–45. doi: 10.1038/s41431-026-02214-7 (PMC13743772; doi:10.1038/s41431-026-02214-7)
Supplement: Supplementary file 1 [file 41431_2026_2214_MOESM1_ESM.docx]

**Supplemental Material**

**Supplemental note: Case reports**

**Individual 1**

This 6-year old female was diagnosed with DLD (age at diagnosis unknown). Her total non-verbal IQ was 83, and she had both fine and gross motor delays. Her height (+1.5 SD) and weight (-0.1 SD) were within the normal range; she had macrocephaly (head circumference +3 SD). Otherwise, she had no significant comorbidities. No evident facial dysmorphisms were observed. Her first-degree family history was unremarkable. Trio-based genome sequencing showed a pathogenic *de novo* missense variant in *NFIX*, which is associated with Malan syndrome (OMIM #614753)[1-3] and fitted the girl’s phenotype well. We referred the child to a pediatrician specialized in genetic disorders for follow-up, and advised a one-time cardiac ultrasound and ophthalmological examination. The recurrence risk for possible future offspring is 50%.

**Individual 2**

This 9-year old boy was diagnosed with DLD at the age of ~5 years. His total non-verbal IQ was 86, and he had no specific behavioral difficulties. He was diagnosed with eosinophilic esophagitis for which he used omeprazole and was under care of a pediatrician; he also had recurrent ear infections. His growth parameters were normal (height 0 SD, weight +0.5 SD, head circumference -1.1 SD). He had no evident facial dysmorphisms. His first-degree family history was unremarkable. Trio-based genome sequencing showed compound-heterozygous pathogenic variants in *PAH*, and a *de novo* 6q14.3 microduplication of ~900 kb (VUS). The boy was referred to a pediatrician specialized in metabolic diseases within our hospital, who conducted additional metabolic tests and diagnosed him with mild phenylketonuria (PKU) or hyperphenylalaninemia (HPA) (OMIM #261600). The tests revealed increased values of phenylalanine (246-384 µmol/L, normal range 36-104 µmol/L), which were currently below the threshold for treatment. Since his phenylalanine levels at a younger age were unknown, the causal relationship between this genetic pathogenic finding and the DLD was not entirely certain. He will stay under follow-up of the pediatrician. It was discussed with parents that the boy’s asymptomatic siblings could be tested for their phenylalanine levels.

**Individual 3**

This 14-year old boy with DLD (age at diagnosis unknown) received ambulatory (Cluster 2) support in the past and temporarily went to a special education school. His total non-verbal IQ was 95, and he had a gross motor delay. According to his parents, he showed some signs of ASD (but has no official diagnosis) and had concentration problems. Neonatally, he was diagnosed with an inguinal hernia and unilateral cryptorchidism; he also had a mild scoliosis. His growth parameters were normal (height -0.3 SD, weight +0.4 SD, head circumference +1 SD). There were no evident facial dysmorphisms. His first-degree family history was unremarkable, besides stuttering and self-reported signs of ASD in father. Trio-based genome sequencing identified a pathogenic *de novo* 16p11.2 microdeletion, which was compatible with his clinical presentation (OMIM #611913). We discussed the result with the affected individual and his parents, and offered them the option to visit to the national expertise center for 16p11.2 microdeletions. The recurrence risk for the affected individual’s possible future offspring is 50%.

**Individual 4**

This 11-year old female was diagnosed with DLD at the age of ~3 years. She had a total IQ of 76. She had a gross motor delay, hypotonia, sleeping problems and recurrent ear infections. She was tall (height +2 SD), had a low-normal weight (-1.7 SD) and microcephaly (head circumference -2 SD). She had mild facial dysmorphisms, including telecanthus, a thin upper lip and microretrognathia. Her first-degree family history was unremarkable. Trio-based genome sequencing identified a pathogenic *de novo* 2p16.3 microdeletion, including *FBXO11* (OMIM #618089; associated with NDD, and aligning with her phenotype) and *MSH6* (OMIM #614350; linked to Lynch syndrome). She was referred to a pediatrician specialized in genetic disorders for follow-up care, and to a clinical geneticist with expertise in cancer genetics. The Lynch-related medical screening program will be initiated at age 25. The girl has a 50% chance of passing the variant to her possible future offspring.

**Individual 5**

This 11-year old girl was diagnosed with DLD at the age of ~3 years. Her total non-verbal IQ was 77, she was sensitive to stimuli and had difficulties in social situations. She had no significant comorbidities. Her growth parameters were normal (height +0.1 SD, weight +0.6 SD, head circumference -0.2 SD). No evident facial dysmorphisms were observed. Her first-degree family history was unremarkable, besides a mild speech delay in her brother. Trio-based genome sequencing showed triple X syndrome, which can cause a variable neurodevelopmental disorder[4] and was compatible with her features. We discussed the genetic result with parents and informed them about a national center with expertise in X and Y trisomies.

**Individual 6**

This 12-year old girl with DLD (age at diagnosis ~9 years) had a total non-verbal IQ of 96, and she had been diagnosed with ADHD. She had a mild pectus excavatum, hypermobility, hypotonia and had sleeping difficulties in the past. Her growth parameters were within the normal range (height -0.3 SD, weight -1.1 SD, head circumference -1 SD). There were no evident facial dysmorphisms. Her father and brother also had ADHD. Trio-based genome sequencing revealed a likely pathogenic *de novo* missense variant in *TRRAP* (OMIM #618454), that could likely explain her phenotype. She was referred to a pediatrician specialized in genetic disorders for follow-up. Also, a one-time cardiac ultrasound was advised. The recurrence risk for the girl’s possible future offspring is 50%.

**Individual 7**

This 12-year old girl was diagnosed with DLD at the age of ~6 years. She had a total non-verbal IQ of 79 and suffered from anxiety with concentration problems in the past. Her height (-0.42 SD) and head circumference (-1.5 SD) were within the normal range; her weight was low (weight-for-height -3.6 SD). Her first-degree family history was unremarkable. Mild facial dysmorphisms were observed, including a long and narrow face, arched eyebrows, a prominent nasal bridge and a small chin. Trio-based genome sequencing revealed a likely pathogenic *de novo* truncating variant in *TAF4* (OMIM #620450), which was likely compatible with her clinical features. She also had a *de novo* truncating variant in *FBRSL1* (VUS). The girl was already under care of a pediatrician for her underweight. We informed the parents and pediatrician about the genetic findings and suggested follow-up by the pediatrician and a one-time cardiac ultrasound. There is a 50% chance of passing the likely pathogenic variant to her possible future offspring.

**Individual 8**

This 5-year old girl with DLD (age at diagnosis ~2-3 years) had a total non-verbal IQ of 95. She was sensitive to stimuli with occasional temper tantrums. She had no significant comorbidities. Her height (-1.5 SD) and head circumference (-1.8 SD) were within the low-normal range; she was slightly overweight (weight-to-height +2.2 SD). Mild facial dysmorphisms were a prominent upturned nasal tip and a long, smooth philtrum. Her first-degree family history was unremarkable. Trio-based genome sequencing showed a likely pathogenic *de novo* 14q11.2 duplication, which fitted her phenotype. This result was shared with the pediatrician who had been involved in the past. The girl has a 50% chance of passing the genetic variant to her possible future offspring.

**Individual 9**

This 9-year old boy was diagnosed with DLD at the age of ~3.5 years. He had a total non-verbal IQ of 77, and had ADHD. He had recurrent ear infections, for which he had ear tubes. His growth parameters were normal (height +0.87 SD, weight +0.98 SD, head circumference 0 SD). He had no evident facial dysmorphisms. His first-degree family history was unremarkable. Trio-based genome sequencing revealed two microduplications: a *de novo* 10p15.3 microduplication (VUS), and a maternal 2q13 microduplication (likely pathogenic). The latter is viewed as a risk factor for developmental delay and learning difficulties.[5] The recurrence risk for this variant is 50%.

**Individual 10**

This 4-year old girl was diagnosed with DLD at the age of ~3 years. She had a total non-verbal IQ of 112. She had recurrent ear infections and rhinitis, but no significant other comorbidities. Her growth parameters were normal (height -1 SD, weight +0.9 SD, head circumference -0.4 SD). Mild facial dysmorphisms included a flat midface and small mouth. Her first-degree family history was unremarkable, besides a mild speech delay in her brother. Trio-based genome sequencing revealed a suspicious truncating variant in *CHD9*. This gene is not an OMIM-disease gene yet (and therefore this variant is reported as a VUS), but has been reported in relation to NDD.[6, 7] The variant was submitted to GeneMatcher.

**Individual 11**

This 5-year old boy was diagnosed with DLD at the age of ~5 years. His total non-verbal IQ was 93, and he had a motor delay. He had concentration problems and was sensitive to stimuli. He had a congenital venous malformation on his lip and various hyperpigmentations and hypopigmentations. He had a small stature (-2.1 SD), normal weight (-1.3 SD) and microcephaly (-3.9 SD). He had mild facial dysmorphisms, including telecanthus, mildly downslanted palpebral fissures, a depressed nasal bridge and prominent nasal tip. His first-degree family history was unremarkable, besides ADHD in mother. Trio-based genome sequencing according to our study protocol did not reveal abnormalities. However, this patient simultaneously received trio-based exome sequencing in a clinical setting, which revealed compound-heterozygous variants in *BRCA2* leading to Fanconi anemia type D1. Chromosome breakage analysis was abnormal, confirming the diagnosis and pathogenicity of the variants. He was referred to a pediatric oncologist for recommended medical care and follow-up. His sister was also tested with chromosome breakage analysis, which was normal. His parents were both heterozygous for one pathogenic *BRCA2* variant (with reduced penetrance for the maternal variant) and were referred to a clinical geneticist with expertise in cancer genetics.

**Figure S1:** Inclusion of participants

**Figure S2: IGV screenshots for reported CNVs/SVs**
Individual 2 – 6q14.3 dup (dn)

Individual 3 – 16p11.2 del (dn)

Individual 4 – 2p16.3 del (dn)

Individual 5 – Triple X

Individual 8 – 14q11.2 dup (dn)

Individual 9 – 2q13q14.1 dup (mat)

Individual 9 – 10p15.3 dup (dn)

**References**

1.	Priolo, M., et al., *Further delineation of Malan syndrome.* Hum Mutat, 2018. **39**(9): p. 1226-1237.

2.	Macchiaiolo, M., et al., *A deep phenotyping experience: up to date in management and diagnosis of Malan syndrome in a single center surveillance report.* Orphanet J Rare Dis, 2022. **17**(1): p. 235.

3.	Klaassens, M., et al., *Malan syndrome: Sotos-like overgrowth with de novo NFIX sequence variants and deletions in six new patients and a review of the literature.* Eur J Hum Genet, 2015. **23**(5): p. 610-5.

4.	Otter, M., C.T. Schrander-Stumpel, and L.M. Curfs, *Triple X syndrome: a review of the literature.* Eur J Hum Genet, 2010. **18**(3): p. 265-71.

5.	*2q13 recurrent region (distal) (includes BCL2L11)*. 2018 [cited 2025 July 29]; Available from: <https://search.clinicalgenome.org/kb/gene-dosage/region/ISCA-37496>.

6.	Álvarez-Mora, M.I., et al., *Diagnostic yield of next-generation sequencing in 87 families with neurodevelopmental disorders.* Orphanet J Rare Dis, 2022. **17**(1): p. 60.

7.	Cardoso, A.R., et al., *Genetic Variability of the Functional Domains of Chromodomains Helicase DNA-Binding (CHD) Proteins.* Genes (Basel), 2021. **12**(11).
